# Gallium‐Doping Effects on Structure, Lithium‐Conduction, and Thermochemical Stability of Li_7‐3*x*_Ga_*x*_La_3_Zr_2_O_12_ Garnet‐Type Electrolytes

**DOI:** 10.1002/cssc.202100526

**Published:** 2021-05-13

**Authors:** Nancy Birkner, Changlong Li, Shanna L. Estes, Kyle S. Brinkman

**Affiliations:** ^1^ Department of Materials Science and Engineering Clemson University Clemson SC 29634 USA; ^2^ Department of Environmental Engineering and Earth Sciences Clemson University Anderson SC 29625 USA

**Keywords:** batteries, calorimetry, electrolytes, gallium doping, thermochemistry

## Abstract

One of the most promising electrolytes for all‐solid‐state lithium batteries is Li_7_La_3_Zr_2_O_12_. Previously, their thermodynamic stability, Li‐ion conductivity, and structural features induced by Ga‐doping have not been empirically determined or correlated. Here, their interplay was examined for Li_7−3*x*_Ga_*x*_La_3_Zr_2_O_12_ with target *x*Ga=0, 0.25, 0.50, 0.75, and 1.00 atoms per formula unit (apfu). Formation enthalpies, obtained with calorimetry and found to be exothermic at all compositions, linearly decreased in stability with increased *x*Ga. At dilute *x*Ga substitution, the formation enthalpy curve shifted stepwise endothermically, and the conductivity increased to a maximum, coinciding with 0.529 Ga apfu. This correlated with percolation threshold analysis (0.558 Ga apfu). Further substitution (0.787 Ga apfu) produced a large decrease in the stability and conductivity due to a large increase in point defects and blocked Li‐migration pathways. At *x*Ga=1.140 apfu, a small exothermic shift was related to defect cluster organization extending the Li hopping distance and decreased Li‐ion conductivity.

## Graphical abstract

**How much dopant**? This is the first work to correlate Li‐ion conductivity and diffusion coefficients, Li‐vacancies, Ga‐doping, and experimental thermochemistry in Ga‐doped LLZOs. Low concentration doping produces defects that promote conductivity and stepwise endothermic formation enthalpy changes. Concentrated dopant yields poor conductivity and increased defect formation (endothermic shift). Further, an increase results in defect clustering (exothermic contributions).

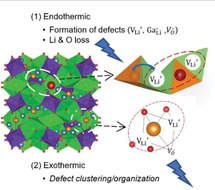

## Introduction

Crystalline lithium lanthanum zirconate Li_7_La_3_Zr_2_O_12_ (LLZO) garnet‐type electrolytes are of interest for all‐solid‐state lithium‐ion batteries due to their high lithium‐ion conductivity on the order of 10^−3^ cm s^−1^ at ambient temperature,^[1]^ low electrical conductivity at room temperature,^[2]^ wide electrochemical window,^[3]^ and high chemical stability at the interface between the garnet electrolyte and the anode Li, which can prevent the occurrence of side reactions.^[4]^

Traditionally, garnets are naturally occurring minerals based on orthosilicates with the general formula A_3_B_2_(SiO_4_)_3_ in which A and B represent eight‐ and six‐coordinated cation sites, respectively, crystallizing in a face‐centered cubic structure (*Ia‐*3*d*). An “ideal” garnet contains a mixture of cations (e. g. metals, rare earth, or post‐transition metals) occupying square antiprismatic, octahedral, and tetrahedral sites with a content stoichiometry of 3 : 2 : 3 to form the general formula A_3_B_2_Si_3_O_12_. Garnet‐like electrolytes are made by replacing silicon to produce the general formula A_3_B_5_O_12_ in which A is substituted with metals or rare earth elements and B with transition or post‐transition metals. Through partial replacement of cations of A or B with higher or lower oxidation state, the lithium content of the structure may be modified, and the structure transformed, both of which affect conductivity.

Doping of a garnet electrolyte can occur on the Li‐, La‐, or Zr‐site, depending on the dopant element. Multivalent dopants transform the undoped tetragonal structure to cubic by substitution into Li‐sites. Lithium sites can be doped with Al,^[5]^ Ga,^[6]^ Fe,^[7]^ and Ge.^[8]^ The use of Ga as Li‐site dopant has been reported to improve Li‐ion conductivity about an order of magnitude (2.6×10^−4^ to 1.2×10^−3^ S cm^−1^) greater than that of Al, as the ionic radius of Ga^3+^ compared to that of Al^3+^ is larger in four‐ and six‐coordination conditions thereby expanding the tetrahedral and octahedral gap.^[9]^ Yet, the exchange of a relatively larger multivalent cation, such as Ga, into Li sites is thought to decrease the length of lithium migration pathway by contraction of the solid‐phase structure, thereby enhancing ion transport through the structure.[^10^, ^11^, ^12^] Thus, there may be a balance between dopant cation size and structural optimization of Li‐ion conduction. Additionally, due to the disruption of the long‐range order of Li‐site occupancy, Li vacancies are produced, which improves Li‐ion transport. Moreover, it has been demonstrated that Li‐ion conductivity is optimized when a content of 6–7 Li atoms per formula unit (apfu) is achieved.[^10^, ^13^, ^14^] A very high Li‐ion conductivity of 1.46 mS cm^−1^ at 25 °C was achieved for Li_7–3*x*_Ga_*x*_La_3_Zr_2_O_12_ at *x*Ga=0.25 and Li=6.25 apfu.^[13]^ Thus, Li‐ion and Li vacancy content play an important role in optimal conductivity.

**Figure 1:**
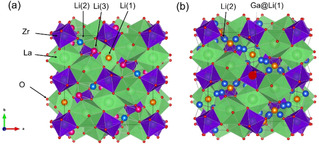
Space‐filling polyhedral illustrations of LLZOs. (a) Undoped LLZO crystallized in a tetragonal crystal system (SG *I*4_1_ */acd*). (b) Ga‐doped LLZO in a cubic crystal system (SG *Ia‐*3*d*) shown in the [001]. Oxygen atoms are represented by small red balls. Available Li sites are signified by the pink, blue, and orange balls in the tetragonal structure. In (b), the dopant gallium atom, shown here as substituted at a Li(1) site, is denoted by a large, dark red ball. The octahedral‐ and dodecahedral‐coordinated metal‐oxygen representations are Zr (purple) and La (green).

A polyhedral rendering of the undoped LLZO tetragonal structure (Figure 1a) shows the ZrO_6_ (Wyckoff position 26c) octahedral sites and LaO_8_ (24d) dodecahedral sites comprising the crystal framework. Here, Li atoms are located at the tetrahedral Li(1) 8a sites (orange) and the distorted sites of Li(2) 16 f (blue) and Li(3) 32 g (pink) in the tetragonal structure. By comparison, in the Ga‐doped cubic structure (Figure 1b), Li atoms are located at the tetrahedral Li(1) 24d (orange) and distorted octahedral Li(2) 96 h (blue) sites. In the Ga‐doped LLZO, Ga (large, dark red ball) substitutes into Li(1) sites.

Crystallographic Li sites in an undoped tetragonal LLZO tend to be fully occupied. Conversely, the Ga‐doped LLZO cubic structure does not fully occupy the Li(1) or Li(2) sites, creating point defects. The resulting distortion assists in developing Li vacancies, which promote the Li‐hopping mechanism crucial to Li‐ion conduction. Li‐site distortion and Li vacancy formation have been considered the structural origin of increased conductivity of Ga‐LLZO relative to undoped LLZO.

Prior work has improved Li_7_La_3_Zr_2_O_12_ and Li_7−3*x*_Ga_*x*_La_3_Zr_2_O_12_ synthesis techniques,[^15^, ^16^, ^17^] structure analysis,^[18]^ as well as the application of theoretical modeling^[19]^ toward maximizing Li‐ion conductivity and a better understanding of the relationship between structure and function. Unfortunately, the correlation of Li‐ion conductivity, dopant content, and point defects is not straightforward. Point defects impact phase stability, thus the relationship between structure, doping level, and ion conductivity should be relatable through thermochemical stability. To that end, interest has increased over the past decade to gain information on the structure–function‐stability interplay of solid‐electrolyte ion conductors.

High‐temperature oxide melt solution calorimetry is capable of rapid and reproducible measurement of refractory material reactions, making this method valuable in the direct measurement of formation enthalpy of garnet‐type electrolytes. Examples of solid‐state electrolytes studied in this way include perovskite‐structured lithium lanthanum titanate (LLTO)^[20]^ as well as the fluorite‐structured yttria‐stabilized zirconia (YSZ)^[21]^ and yttria‐doped barium zirconate (BZY).^[22]^ A general trend is emerging from this body of work suggesting that the phase stability among solid electrolytes decreases with increasing Li‐ion conductivity, which corresponds with increased dopant content. However, the relationship is not continuous but moves toward a maximal Li‐ion diffusion with a limiting dopant content prior to structural changes that diminish Li‐ion conductivity. In other words, with elevated dopant arises a condition of diminishing returns in the economy of structure, such that too much dopant appears to block Li‐migration pathways.[^23^, ^24^, ^25^] Further examination of the complex interplay between structure, conductivity, and thermodynamics will shed more light on these advanced materials.^[26]^

Empirically measured thermodynamic stability and room‐temperature Li‐ion conductivity of Li_7_La_3_Zr_2_O_12_ and Ga‐doped Li_7–3*x*_Ga_*x*_La_3_Zr_2_O_12_ powders have not been previously correlated. For the first time, the enthalpy of formation is obtained for a suite of Li_7–3*x*_Ga_*x*_La_3_Zr_2_O_12_ samples with target Ga content ranging from *x*Ga=0 to 1.00. Stability relationships are explored with respect to Li‐ion conductivity and diffusion coefficients, Li‐vacancies, and Ga‐dopant content.

## Results and Discussion

**Figure 2:**
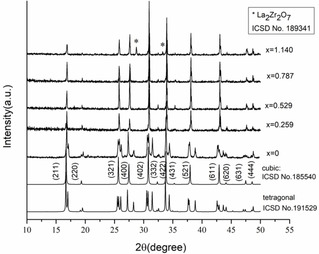
PXRD of the Li_7‐3*x*_Ga_*x*_La_3_Zr_2_O_12_ (*x*Ga=0, 0.259, 0.529, 0.787, 1.140) samples. Reference phases correspond with tetragonal (ICSD 191529) and cubic (ICSD 185540) LLZO structures. Peaks identified for the secondary phase (La_2_Zr_2_O_7_) in sample *x*=1.140 correspond with the pyrochlore reference phase ISCD 189341, which are indicated (*).

Garnet‐type Li_7‐3*x*_Ga_*x*_La_3_Zr_2_O_12_ electrolytes were produced at high temperature (950 °C) with target *x*Ga=0, 0.25, 0.50, 0.75, and 1.00 apfu. The powder X‐ray diffraction (PXRD) peak intensities were calculated, and the lattice parameters were refined. The successive dichotomy method^[27]^ was used to calculate and fit the measured peak intensities (Dicvol06 embedded in Match! 3.0, Crystal Impact). The crystallographic structure data are in Table 1 and PXRD patterns in Figure 2. The undoped structure crystallized in the tetragonal crystal system (SG *I*4_1_ */acd*, No. 142), in agreement with collection code 191529.^[28]^ The Ga‐doped LLZO samples were assignable to cubic crystal system (SG *Ia‐*3*d*, No. 230), corresponding well with ICSD collection code 185540.^[29]^ Contention over Ga site occupancy contributions to Li‐ion conduction highlighted the possibility that Ga‐doped LLZO may belong to a different cubic SG than *Ia‐*3*d*, namely, *I‐*43*d*. Although PXRD identified all Ga‐doped LLZOs as SG *Ia‐*3*d*, it is limited in its ability to discern between the cubic SG *Ia‐*3*d* and *I‐*43*d*. However, single‐crystal (SC)‐XRD and neutron scattering can distinguish between these two cubic space groups. For instance, at *x*Ga≥0.15 apfu in Ga‐LLZO, SG *I‐*43*d* (No. 220) was reported from single crystals using SC‐XRD and neutron diffraction.[^18^, ^30^, ^31^] Due to the limitation of PXRD, there is some uncertainty as to which of these two space groups the Ga‐doped LLZOs belong. Regardless, as all *x*Ga≥0.15 apfu have been reported to belong to the same space group (*Ia‐*3*d* or *I‐*43*d*), we contend that the Ga‐LLZOs of the present study likewise correspond. A pyrochlore secondary phase, La_2_Zr_2_O_7_ (7 %), which is a Li‐ion non‐conductive solid,^[30]^ was identified in the most concentrated sample, *x*Ga=1.140 apfu. A previous report found that the garnet‐type structure was stable up to *x*Ga=1.0 apfu.^[16]^ In this study, all samples were treated with the same procedure, to examine products in which the Ga reactant was the only variable synthesis parameter. Thus the use of additional lithium to shift the synthesis by‐product (La_2_Zr_2_O_7_) in the concentrated gallium sample toward LLZO was avoided.

**Table 1:** Target and ICP‐MS compositions. Crystal systems, calculated lattice parameters, and unit cell volumes. Room temperature Li‐ion conductivity and diffusion coefficients of the as‐synthesized electrolyte powders along with comparable benchmark compositions.

Target composition	ICP‐MS composition	Crystal system	Lattice parameter [Å]	Unit cell volume [Å]	Conductivity^[a,b]^ [mS cm^−1^]	Conductivity Ref.	Diffusion coefficient [cm^2^ s^−1^]	Diffusion coefficient Ref.
Li_7.00_La_3_Zr_2_O_12_	Li_7.90_La_3.0_Zr_1.72_O_11.89_	tetragonal	*a*=13.145(5) *c*=12.638(7)	2183.7330	6.78×10^−4^	this work	4.35×10^−12^	this work
Li_6.25_Ga_0.25_ La_3_Zr_2_O_12_	Li_7.11_Ga_0.259_La_3.0_Zr_1.75_O_11.94_	cubic	12.951(6)	2172.5524	1.65×10^−2^	this work	1.90×10^−9^	this work
Li_5.5_Ga_0.50_ La_3_Zr_2_O_12_	Li_6.12_Ga_0.529_La_3.0_Zr_1.73_O_11.82_	cubic	12.940(8)	2167.1221	5.80×10^−2^	this work	3.27×10^−9^	this work
Li_4.75_Ga_0.75_La_3_Zr_2_O_12_	Li_5.23_Ga_0.787_La_3.0_Zr_1.75_O_11.79_	cubic	12.926(1)	2159.7453	8.08×10^−3^	this work	2.91×10^−10^	this work
Li_4_Ga_1.00_La_3_Zr_2_O_12_	Li_4.89_Ga_1.140_La_3.0_Zr_1.67_O_11.98_	cubic	12.916(5)	2154.9368	4.68×10^−3^	this work	1.05×10^−10^	this work
Li_7_La_3_Zr_2_O_12_	NA	tetragonal	*a*=13.134(4) *c*=12.663(8)	2184.3923	≈1.63×10^−3^ (bulk)	[37]	6.4×10^−12^	[38]
Li_5.5_Al_0.15_La_3_Zr_2_O_12_	Li_6.46_Al_0.15_La_3_Zr_1.95_O_11.86_	cubic	12.970(9)	2182.7793	8.3×10^−2^	[39]	NA	not meas.
Li_6.5_La_3_Zr_1.6_Ta_0.4_O_12_	NA	cubic	NA	NA	4.7×10^−1^ (pellet)	[40]	2.35×10^−9^	[41]
Li_6.5_La_3_Zr_1.5_Nb_0.5_O_12_	NA	cubic	NA	NA	8×10^−1^ (bulk) (dense pellet)	[42]	1.7×10−9 (32 °C)	[43]

[a] Conductivity reported for (bulk) is for the bulk region of the material, (pellet) refers to the measurement of pellet form of the sample. If not explicitly pointed out, it refers to conductivity for pressed powders of the sample. [b] Conductivities and diffusion coefficients of the current work and other reports were measured at 25 °C, unless otherwise noted.

**Figure 3:**
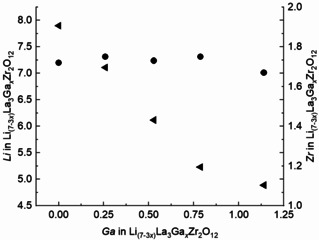
Comparison of lithium (left axis, left‐facing solid arrow) and zirconium (right axis, solid circle) as a function of gallium content in the Li_7‐3*x*_Ga_*x*_La_3_Zr_2_O_12_ samples.

Metal content was measured with inductively coupled plasma mass spectrometry (ICP‐MS). Oxygen content was computed from electroneutrality.[^31^, ^32^] Sample compositions are shown in Table 1. Figure 3 compares the relative Li‐, Zr‐, Ga‐contents. Compositions are stoichiometric within experimental error, with actual content *x*Ga=0, 0.259, 0.529, 0.787, 1.140 apfu. Composition analysis indicates that excess lithium carbonate during synthesis compensated for lithium loss at elevated temperature. Li content decreases linearly with increasing Ga content. The Zr/La molar ratios are the same within experimental error over the range of samples up to *x*Ga=0.787 apfu, with a difference of less than 1.15 %, with that of sample *x*Ga=1.140 apfu it is 4.57 %. Thus, for sample *x*Ga=1.140 apfu, the content of Zr is slightly lower and that of Li slightly higher than expected, which is attributed to the 7 % La_2_Zr_2_O_7_ pyrochlore secondary phase affecting overall structure and composition. The measured Zr deficit is a curious result, although decreased Zr content relative to target Ga‐LLZO composition has been reported elsewhere.[^33^, ^34^, ^35^, ^36^] All reagent oxides (Li_2_CO_3_, La_2_O_3_, ZrO_2_, and Ga_2_O_3_) were verified as phase‐pure prior to synthesis. Likewise, no additional Zr‐containing phases were identified from the PXRD patterns for all compositions, other than that reported for the high concentration sample (*x*=1.14). There may have been an amorphous Zr phase undetectable with PXRD. However, composition analysis diametrically opposed this possibility. The consistency of the Zr mole ratios across the samples (with the exception of the product with the highest Ga content, as discussed above) determined from the ICP‐MS analyses indicated that insoluble Zr phases were not responsible for the Zr deficit. At this time, further comment on this issue is speculative. Regardless of the Zr deficit, the observed trends in conductivity and phase stability as a function of Ga content and structure changes remain intact.

**Figure 4:**
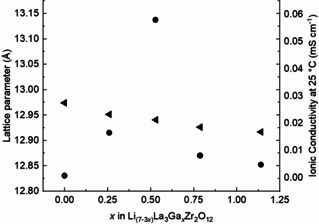
Calculated lattice parameter “*a*” (left axis, left‐facing arrow) and Li‐ion conductivity at 25 °C (right axis, solid circle) of the as‐synthesized powders against *x*Ga in Li_7–3*x*_Ga_*x*_La_3_Zr_2_O_12_ (*x*=0, 0.259, 0.529, 0.787, 1.140 apfu). Because the *x*=0 phase has a tetragonal symmetry, its geometric mean “*a*” parameter was plotted as the cubic root of the unit cell volume to facilitate its comparison with the “*a*” parameter of the other compositions.

As shown in Table 1 and Figure 4, with increases in *x*Ga content, the doped Ga‐LLZO lattice parameters decrease linearly, while the significant differences in Li‐ion conductivity vary nonlinearly. Lattice parameter “*a*” decreases with increasing Ga (Figure 4) and tracks from undoped LLZO to *x*=1.140 Ga LLZO, as 13.1455>12.9516>12.9408>12.9261>12.9165 (Å). The largest decrease (0.1939 Å) is due to an initial incorporation of Ga (*x*=0.259 apfu). Unit cell volume changes with a lattice parameter, related here to bond length decrease and lowering of tetragonal distortion to form the cubic structure. The unit cell volumes decrease linearly with increasing *x*Ga incorporation into the LLZO structures, corresponding as 2183.7330>2172.5524>2167.1221>2159.7453>2154.9368 (Å), as shown in Table 1.

As reported previously, due to charge compensation,^[44]^ incorporation of one Ga^3+^ ion into the structure displaces one Li^+^ ion at a Li(1) tetrahedron site and vacates two Li^+^ ions from Li(2) octahedron sites resulting in the formation of two Li vacancies.^[18]^ Due to atomic radius mismatch, substitution of gallium for lithium [*r*(Ga^3+^)=61 pm < *r*(Li^+^)=73 pm] in the 4‐coordinate tetrahedral (24d) site is expected to contribute to lattice distortions.^[45]^ Exchange of increasing amounts of Ga into Li sites is correlated linearly with small decreases in lattice parameters and unit cell volumes resulting in structure contraction.^[35]^ The Ga/Li exchange can be generalized using Kröger‐Vink notations, as in Equation 1:^[46]^

(1) $$Ga_{2}O_{3}\;\overset{Li_{2}O}{\to }2Ga_{\mathrm{Li}}^{\cdot \cdot }+3O_{o}^{x}+4V_{\mathrm{Li}}^{{}^{'}}$$

Conductivity (Table 1 and Figure 4) of the as‐synthesized powders track nonlinearly with the lattice parameter from *x*Ga=0 to 1.140. A steep increase occurs moving from the *x*Ga=0 (7.90 Li) apfu undoped tetragonal structure to that of the cubic structure *x*Ga=0.259 (7.11 Li) apfu. This is followed by an increase to Li‐ion conduction performance maximum at *x*Ga=0.529 (6.12 Li) apfu. At *x*Ga>0.529, a large decrease in Li‐ion conductivity occurs as *x*Ga=0.787 (5.23 Li) apfu is approached, measuring less than that of *x*Ga=0.259 apfu, and surpassed in diminished performance only by *x*Ga=1.140 (4.89 Li) apfu. The samples with the highest concentration of *x*Ga (0.787 and 1.140 apfu) have Li contents lower than 6 Li apfu, reflecting a lack of active Li ions available for the hopping mechanism. Indeed, among the sample suite of this work, *x*Ga=0.529 apfu does fall within the reported “sweet spot” of optimally performing LLZOs corresponding with 6–7 Li apfu.^[13]^ For the *x*Ga=1.140 sample, an additional contribution to decreased conductivity is the small content (7 %) of a non‐conductive secondary phase, pyrochlore (La_2_Zr_2_O_7_), which blocks Li‐ion transport between grain‐boundaries.^[30]^

Generally, the total ionic conductivity of Ga‐doped LLZO pellets can achieve up to 5.81×10^−2^ mS cm^−1^,^[47]^ when pellets are fabricated to possess high relative density and larger grains. Li‐ion conductivity of the present work was performed on as‐synthesized powders and, as expected, the conductivity is lower than previous reports of dense, sintered LLZO pellets.[^13^, ^16^, ^37^] In solid‐state battery applications, LLZOs are prepared in their final form by pressing their powders into pellets and then sintering at a high temperature. This densification process will enhance the relative density, which yields greater Li‐ion conductivity by roughly an order of magnitude.^[47]^ However, processing increases the opportunity for surface contamination. LLZOs are extremely sensitive to reaction with H_2_O and CO_2_, easily forming Li_2_CO_3_ at their surfaces.[^48^, ^49^] As the purpose of the current study is to examine the relationship between composition, performance, and thermodynamics of Ga‐doped LLZOs, the sample powders were thus handled in a dry environment and measured to avoid surface contamination. This precluded their being processed into pellets for Li‐ion conductivity measurement.

Li‐ion conductivity of a solid‐state conductor is determined by the co‐effects between the number of carrier ions and the mobility of active Li ions as shown in the general relation, Equation 2:^[50]^

(2) $$\sigma_{i}=\sum n_{i}eu_{i}$$

Here, the terms *n_i_*, *e*, and *u_i_* correspond with the number of *i*th carrier ions, the charge of an electron, and the mobility of active *i*th Li ions, respectively. Theoretical investigation found that Al incorporation into Li(1) sites (24d) may create “blocking effects” that hinder Li‐ion migration.[^23^, ^24^, ^25^] Substitution of Ga for Li and the subsequent decrease in Li‐ion number increases local crystal distortion. All levels of Ga doping produce blocking of Li migration to some extent. At low *x*Ga content, incorporation into the structure begins the formation of Li vacancies with subtle emergence of Li‐migration pathways not yet pronounced until some optimal *x* is achieved. It seems self‐evident then, at elevated *x*Ga, blocking effects are expected to be more significant, and Li‐ion conductivity then decreased.

Li‐vacancy formation is a crucial factor in the Li hopping mechanism in LLZOs. However, Equation (2) indicates ion mobility suffers when the number of Li vacancies exceeds a certain number. The result of excessive Li vacancy number is the formation of vacancy clusters. Large clusters of Li‐vacancies reportedly extend the Li hopping distance,^[51]^ which increases Li‐ion migration energy barriers.^[52]^ As the dopant level *x* is increased, the distribution of point defects will begin to develop more proximal rather than distal,^[53]^ and as such they may cluster. Thus, at high *x*Ga there is decreased Li‐ion conductivity due to the co‐effect of Li vacancy clustering in which clusters do not contribute to the migration pathway in addition to the Ga “blocking effects”.

**Figure 5:**
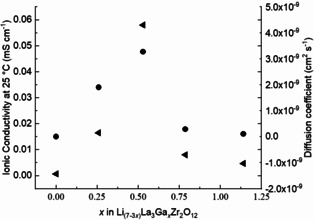
Li‐ion conductivity (left axis, left‐facing arrow) and corresponding diffusion coefficient (right axis, solid circle) against *x*Ga in Li_7−3*x*_Ga_*x*_La_3_Zr_2_O_12_ (*x*=0, 0.259, 0.529, 0.787, 1.140 apfu).

The diffusion coefficient values are shown in Table 1 and Figure 5 along with ionic conductivity with respect to *x*Ga content. For sample *x*Ga=0.529 apfu, the Li‐ion diffusion coefficient at 25 °C (3.27×10^−9^ cm^2^ s^−1^) is approximately three orders of magnitude greater than that of *x*Ga=0, undoped Li_7_La_3_Zr_2_O_12_ (5.26×10^−12^ cm^2^ s^−1^). The temperature‐dependent Li‐ion diffusion coefficient (*D*_*σ*Li+_) can be estimated from the measured Li‐ion conductivity (*σ*_Li+_) and (*N*_NE_) the number of carrier ions (active Li ions). The general relation between *D*_*σ*Li+_ and *σ*_Li+_ is described by the Nernst–Einstein equation^[54]^ [Eq. 3]:

(3) $$D_{{\sigma_{Li}}^{+}}=\frac{\sigma_{Li^{+}}K_{B}T}{N_{NE}\;e^{2}}$$

Here, *K*_B_ is the Boltzmann constant (1.38×10^−23^ J), and *e* is the electron charge (1.60×10^−19^ C). *N*_NE_ is the number of carrier ions, which equals the Li vacancy concentration. It can be calculated by the relationship of volumetric mass density (*ρ*), Avogadro's number (6.022×10^23^ mol^−1^), and Li vacancies per mole formula unit with respect to the molecular weight (*M*_w_) of the sample, as shown in Equation (4).^[43]^ For the tetragonal LLZO, the *N*_NE_ is 2.5×10^22^ cm^−3^.^[38]^ For all cubic Ga‐doped LLZOs, the moles of Li vacancies per formula unit can be obtained from Ga doping concentration and the unit cell volume of each sample.

(4) $$N_{NE\;}=\frac{\rho \times A\times moles\;Li\;vacancy\;p.f.u.\;}{M_{w}}$$

Diffusion is defined by carrier ion concentration and mobility as factors controlling ionic conductivity (*σ*). Optimal ionic conductivity can be evaluated using the limiting relationship of site percolation threshold with respect to the sum of Li‐ion and Li‐vacancy content, which is proportionally related as (*σ*) ∝ (*N*−*N*_c_)^2^.^[55]^ Here, *N* is evaluated as the sum of Li‐ion and Li‐vacancy content, and *N*_c_ is the percolation threshold. When *N* exceeds *N*_c_, then the Li‐ion conduction channels are interconnected through the structure. In the garnet‐type structure, *N* is 7−*x*, which equals sum of Li‐ion and Li‐vacancy content (7−3*x*+2*x*). The threshold, *N*_c_, is 0.529 (this work) for *x* Ga‐doped cubic LLZO garnet, which is larger than that of the simple cubic garnet system (0.3117) reported previously.^[55]^ The effective carrier concentration (*N*_eff_) is computed using Equation 5:

(5) $$N_{eff}\;\propto m\times \left(1-m\right)=\frac{2x\;\times \;\left(7-3x\right)}{{\left(7-x\right)}^{2}}$$

Here, *m* (e. g., 7−3*x*/7−*x*) represents the ratio of Li to the sum of Li‐ion and Li‐vacancy content. When *x* Ga and effective carrier concentration are taken together, they relate the percolation threshold [Eq. (6)] to a maximum conductivity:

(6) $$\sigma \;\propto {m\left(1-m\right)\left(N-N_{c}\right)}^{2\;}=\;\frac{2x\;\times \;\left(7-3x\right)}{{\left(7-x\right)}^{2}}{\left(7-x-N_{c}\right)}^{2}$$

This allows the calculation of a theoretical *x*Ga concentration at maximum conductivity. The concentration at maximum conductivity using percolation threshold analysis occurs for a theoretical sample of *x*Ga=0.558 apfu, which correlates well with the actual sample *x*Ga=0.529 apfu.

**Figure 6:**
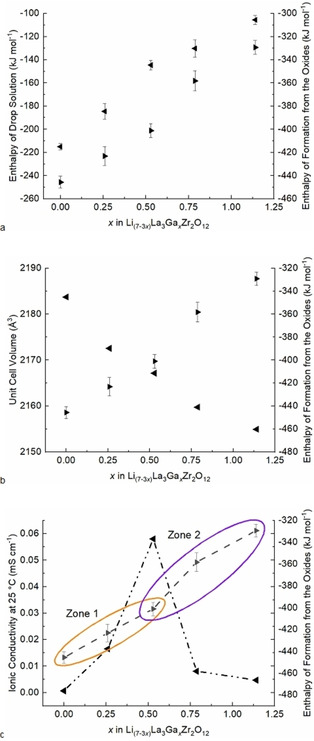
(a) Enthalpies of drop solution (left axis, left‐facing arrow) and formation enthalpies (right axis, right‐facing arrow). The experimental uncertainties are indicated. (b) Unit cell volume (left axis, left‐facing arrow) and formation enthalpies (right axis, right‐facing arrow). (c) Li‐ion conductivity (left axis, left‐facing arrow, dash‐dot‐dot line) and formation enthalpies (right axis, right‐facing arrow, dash‐dash line). Plots (a–c) are with respect to *x*Ga content in the Li_7−3*x*_Ga_*x*_La_3_Zr_2_O_12_ samples.

High‐temperature oxide melt solution calorimetry, at 700 °C in sodium molybdate, was applied to investigate stability relationships of gallium substitution effects in Li_7–3*x*_Ga_*x*_La_3_Zr_2_O_12_. The enthalpies of formation from the oxides (Δ*H*_f,ox_) were calculated from the high‐temperature drop solution enthalpies (Δ*H*_ds_) of component binary oxides (Li_2_O, La_2_O_3_, Ga_2_O_3_, and ZrO_2_) and the samples using appropriate thermochemical cycles (Table 2). Results are shown in Table 3 and Figure 6a–c.

**Table 2:** Thermochemical cycles for Li_(7‐3*x*)_Ga_*x*_La_3_Zr_2_O_12_ that compute the enthalpy of formation from the oxides at 25 °C based on mean enthalpies of drop solution.

Reactions		Δ*H*_ds_^[a]^ [kJ mol^−1^]
(1) Li_(7‐3*x*)_Ga_*x*_La_3_Zr_2_O_12(s, 25 °C)_→(7−3*x*)/2 Li_2_O_(sln, 700 °C)_+ 3/2 La_2_O_3(sln, 700 °C)_+*x*/2 Ga_2_O_3(sln, 700 °C)_+2 ZrO_2(sln, 700 °C)_		Δ*H*_1_ *=*Δ*H*_ds_(Li_(7‐3*x*)_Ga_*x*_La_3_Zr_2_O_12_)
(2) Li_2_O_(s, 25 °C)_→Li_2_O_(sln, 700 °C)_		Δ*H*_2_=−90.3±2.5^[56]^
(3) La_2_O_3(s, 25 °C)_→La_2_O_3(sln, 700 °C)_		Δ*H*_3_=−225.10±3.16^[57]^
(4) Ga_2_O_3(s, 25 °C)_→Ga_2_O_3(sln, 700 °C)_		Δ*H*_4_=130.16±1.66^[58]^
(5) ZrO_2(s, 25 °C)_→ZrO_2(sln, 700 °C)_		Δ*H*_5_=19.5±0.9^[59]^
**Enthalpy of formation from the component binary oxides to form (Li_(7‐3*x*)_La_3_Ga**_***x***_ **Zr_2_O_12_)**:		
(7–3*x*)/2 Li_2_O_(s, 25 °C)_+3/2 La_2_O_3(s, 25 °C)_+*x*/2 Ga_2_O_3(s, 25 °C)_+2 ZrO_2(s, 25 °C)_→Li_(7‐3*x*)_La_3_Ga_*x*_Zr_2_O_12(s, 25 °C)_		
Δ*H*_f,ox_(Li_(7−3*x*)_Ga_*x*_La_3_Zr_2_O_12_)=(7–3*x*)/2Δ*H*_2_+3/2 Δ*H*_3_+x/2 Δ*H*_4_+2 Δ*H*_5_−Δ*H*_1_		

[a] Enthalpies of drop solution are the mean of several experiments with reported uncertainties as two standard deviations of the average value.

**Table 3:** Enthalpy of drop solution (Δ*H*_ds_) and formation enthalpy from the oxides at 25 °C (Δ*H*_f,ox_).

Composition	Δ*H*_ds_^[a]^ [kJ mol^−1^]	Δ*H*_f,ox_ [kJ mol^−1^]
Li_7.90_La_3.0_Zr_1.72_O_11.89_	−215.09±2.64 (8)	−445.71±5.17
Li_7.11_Ga_0.259_La_3.0_Zr_1.75_O_11.94_	−184.53±6.66 (8)	−423.16±8.01
Li_6.12_Ga_0.529_La_3.0_Zr_1.73_O_11.82_	−144.66±4.10 (8)	−401.15±6.05
Li_5.23_Ga_0.787_La_3.0_Zr_1.75_O_11.79_	−130.20±7.36 (8)	−358.24±8.60
Li_4.89_Ga_1.140_La_3.0_Zr_1.67_O_11.98_	−105.67±3.87 (8)	−329.30±5.84^[b]^

[a] Δ*H*_ds_ values are the measured mean from the number of experiments shown in parentheses, and the±uncertainty is the two‐standard deviation in the measurement mean. [b] An enthalpy correction was applied to sample *x*Ga=1.140 to account for the 7 % La_2_Zr_2_O_7_ pyrochlore secondary phase using the enthalpy of formation, Δ*H*_f,ox_=−107.3±5.1 kJ mol^−1^.^[60]^

The enthalpies of drop solution (Figure 6a) are more positive linearly with increased Ga content (decreased Li) indicative of increasing destabilization. The formation enthalpies are strongly exothermic for all LLZO compositions, which indicates thermodynamic stability with respect to their component binary oxides. Additionally, the formation enthalpies become more negative (thermodynamically favorable) linearly with decreasing Ga content (increased Li). Trending toward destabilization with increased Ga, positive enthalpy contributions may be attributed at least partly to lattice distortions as the small radius Ga^3+^ exchanges at one Li(1) site and subsequently creates two Li vacancies. It was previously reported that atomic radii mismatch and lattice strain energy produce a positive enthalpy in solid electrolyte structures.^[20]^

With increased Ga incorporation, the lattice structure volume decreases linearly, and the formation enthalpy trends toward destabilization (Figure 6b). Enthalpy shifts occur along with the formation enthalpy curve with increasing *x*Ga in contrast to the stepwise decrease in unit cell volume. The shifting enthalpy versus a constant change in unit cell volume with Ga increase suggests that the influence of Ga substitution on Ga‐LLZO stability is related to additional structural changes other than the resulting effects of atomic radius substitution mismatch and strain energetics.

Figure 6c compares the dilute and concentrated Ga content regions (zones 1 and 2) for the changes in the formation enthalpy and Li‐ion conductivity against *x*Ga. The formation enthalpy curve illustrates the general trend and the enthalpy shifts in the formation enthalpies, while Table 3 presents the values. Overall, the formation enthalpy curve is linear (*R* ^2^=0.9849) with a general trend that is endothermic with increasing Ga content. Endothermic shifts along the formation enthalpy curve correspond with defect processes that produce positive enthalpy contributions, which include Ga substitution at Li(1) sites, Li loss, Li vacancy formation, lattice distortions, and random ordering/distribution of dopants and vacancies. At concentrated levels of Ga content, the curve presents significant endothermic and exothermic enthalpy shifts attributable to the increased formation of defects. These shifts and their energetic relationship to Li‐ion conductivity and point defects are discussed below.

In the dilute *x*Ga concentration region (Figure 6c, zone 1), at *x*Ga=0–0.529 apfu, the formation enthalpy curve is constant with a stepwise endothermic increase in the formation enthalpy against *x*Ga (Δ*H*_f,ox_=22 kJ mol^−1^). At these lower concentrations of *x*Ga, defect formation, although destabilizing to the structure, also includes the formation of Ga and Li vacancy point defects. Here, the point defects produced by Ga substitution correspond to increasing Li‐ion conductivity observed along 0<*x*=0.259 to a maximum at *x*=0.529, which agrees well with percolation threshold analysis that computed a maximum Li‐ion conductivity value coinciding with *x*Ga=0.558 apfu (see above). Indeed, ion diffusion is optimized when Li migration pathways have spanned the structure, and as such, it is reasonable to assume that Ga blocking effects are not hindering Li‐ion conduction in this more dilute region of Ga substitution. Comparison (Figure 6c, zone 2) of the composition of maximum conductivity (*x*Ga=0.529, Δ*H*_f,ox_=‐401.15 kJ mol^−1^) with the next stepwise increase in *x*Ga concentration (*x*Ga=0.787, Δ*H*_f,ox_=‐358.24 kJ mol^−1^) structural changes emerge that drive a much larger endothermic (positive) shift as demonstrated by a change in formation enthalpy of 43 kJ mol^−1^. Figure 6c (zone 2) illustrates this large formation enthalpy difference as an abrupt change in the curve between these compositions. The enthalpy difference is about twice the change in the formation enthalpy occurring between each preceding composition. Increased concentration in defect formation is thermodynamically destabilizing to the structure and, at *x*Ga=0.787, coincides with significantly decreased Li‐ion conductivity suggesting Ga blocking defects have formed, which interfere with the Li‐hopping mechanism. At the most concentrated *x*Ga content (*x*=1.14, Δ*H*_f,ox_=−329.30 kJ mol^−1^), the direction of the curve is yet overall endothermic, yet the formation enthalpy shift does not progress along the same steep path as that of *x*Ga=0.787 (Figure 6c, zone 2). Instead, the formation enthalpy difference between *x*Ga=1.14 and 0.787 is 29 kJ mol^−1^ indicating exothermic structural changes occur at this composition that competes against the increasingly unfavorable thermodynamics of defect formation at concentrated Ga compositions. The exothermic contribution is attributable to defect organization, such as Ga substituted Li(1) sites and Li vacancies, which extends the Li‐ion hopping distance and decreases Li‐ion conductivity. Development of the two main exothermic defect processes focused on in this work begins with Ga^3+^ substitution on Li(1) sites resulting in the addition of +2 charge in the local region concurrent with the loss of two nearby Li^+^ ions to form Li vacancies (*V′*_Li_) and a local −2 charge. As detailed in the 2021 computational paper by Li et al.,^[53]^ Ga substitutions prefer to occur more proximal than distal in the structure, likewise, the two departing Li ions are near neighbors to the substituted Ga. As such, with increased concentration of such thermodynamically destabilizing development of defects, it stands to reason that a large endothermic shift in stability would be observable. Likewise, if subsequent thermodynamically stabilizing organization or association of defects occurred, then an exothermic shift in stability would be observable. These former and latter energetic shifts are prevalent in zone 2, respectively. An alternative likelihood is defect association between the negatively charged *V*_Li_ and positively charged *V*_ö_ to form Schottky defect pairs. To that end, a resulting *V*_Li_–*V*_ö_ defect association is also energetically favorable (exothermic) relative to a high concentration of Li vacancies. Both of the two exothermic defect processes are energetically favorable, contributing to the decrease in the formation enthalpy curve steepness in zone 2. Likewise, additional point defects are potential sources of endothermic shifts.^[61]^ Indeed, the point defects described above are the principal defects considered in this work as they are more thermodynamically probable processes in garnet‐type solid electrolytes (LLZOs) than those produced by Zr, La, or O, according to DFT studies^[53]^ related to this work.

The thermochemical trend in the LLZO samples indicates that their energetics do not change solely as a function of Ga incorporation (Li loss and Li‐vacancy formation). Although structurally organized clustering of dopants and vacancies are thermodynamically favorable (exothermic), as is increased Li content, in terms of the Li‐migration through the structure, these contributions appear to have limitations or diminishing returns on Li‐ion conductivity. Some Li migration may be hindered at any level of Ga content, whereas elevated *x*Ga content is expected to significantly decrease Li‐ion conductivity due to the co‐effect of Li vacancy clustering and Ga “blocking effects.” As discussed above, when the sum of Li ion content and Li vacancy exceeds the percolation threshold, which is defined as the garnet LLZO dopant content *x*, then the Li‐ion migration pathways are interconnected through the structure. In this case, conductivity is optimized. The interplay between Ga‐doped LLZO defects and conductivity is readily interpretable using formation enthalpies. Indeed, such dopant‐structure thermodynamic tendencies were reported previously for solid electrolyte structures including, YBZ proton conductors,^[22]^ LLTO Li‐ion conductors,^[20]^ fluorite oxide ion conductors,^[62]^ and perovskite proton conductors La_*x*_Th_1‐x_O_2‐0.5x_.^[63]^ However, the exact measurement of internal defects, which delineates their organization within the structure, would further reveal the underpinnings of the structure–thermochemistry relationship and influence on Li‐ion conductivity.

## Conclusion

High‐temperature oxide melt solution calorimetry was used to obtain the enthalpy of formation from the oxides of Ga‐doped Li_7_La_3_Zr_2_O_12_ (LLZO) garnet‐type electrolytes for the first time and demonstrates highly stable formation enthalpies (exothermic) relative to their binary oxide reaction components (Li_2_O, La_2_O_3_, Ga_2_O_3_, and ZrO_2_). Increased Ga content resulted in an endothermic contribution that destabilizes the garnet structure through the formation of defects. Point defects contribute to changes in the thermodynamic stability as well as to the ionic conductivity. The Ga‐LLZO of maximum conductivity (0.529 Ga apfu) is thermodynamically destabilized relative to samples of more dilute Ga content (0 and 0.259 apfu). Likewise, it is more thermodynamically stable than samples more concentrated in Ga content (0.787 and 1.140 apfu), which consequently are also much lower in conductivity. This significant shift in destabilization corresponding with prominent decrease in conductivity occurs in the region just above dilute Ga content, which is due to increased formation of defects that also block Li conductivity. Percolation threshold analysis of the maximum Li diffusion through the Ga‐doped LLZO garnet structures was optimized for Ga content of *x*Ga=0.558 apfu. At this level of Ga‐dopant content, the Li‐ion and Li‐vacancy contents were optimized for maximum conductivity, achievable through the development of crucial Li migration pathways that span the structure. The nearest composition measured in the current work was *x*Ga=0.529 apfu, which did exhibit maximum conductivity relative to all other compositions across *x*Ga=0, 0.259, 0.529, 0.787, 1.14 apfu. Additionally, the Li content of sample *x*Ga=0.529 (6.12 Li) apfu correlated well with the reportedly optimal Li content for the Li‐hopping mechanism crucial to Li‐ion conductivity. Understanding the relationships among structure, conductivity, and thermodynamic stability of various site‐substituted LLZOs and identifying general trends among LLZOs is fundamental and applies to the advanced material design of Li‐ion conductors for all‐solid‐state Li‐ion batteries.

## Experimental Section

### Materials

Commercial precursor powders of Li_2_CO_3_ (99.9 %, Sigma‐Aldrich), La_2_O_3_ (99.99 %, Acros Organics), ZrO_2_ (99 %, Sigma‐Aldrich), and Ga_2_O_3_ (99.99 %, Inframat) were used as received. Just prior to use, the precursor La_2_O_3_ and ZrO_2_ powders were dried at 900 °C (2 h) to remove water and carbonate. Li_2_CO_3_ was dried at 200 °C (overnight) to remove water.

### Synthesis

Undoped LLZO (Li_7_La_3_Zr_2_O_12_) and Ga‐doped LLZO (Li_7‐3*x*_Ga_*x*_La_3_Zr_2_O_12_, with target *x*Ga=0, 0.25, 0.50, 0.75, 1.00 apfu) were synthesized from precursor powders at stoichiometric ratios using conventional solid‐state reaction at 950 °C. Excess (10 wt %) Li_2_CO_3_ was added in each preparation to compensate for lithium loss due to the volatilization of lithium oxide at high temperatures. More specifically, the preparation method entailed ball‐milling (rolling, 48 h) of reaction powders in a 300 mL screwcap Nalgene bottle containing 150 mL 99 % pure acetone (Xtractor‐Depot®) with 6 and 12.5 mm zirconia balls. Homogeneity of the mixing environment was improved using acetone as the grinding solvent due to its polar and nonpolar nature. Resulting mixtures were then placed under infrared light (200 °C, ≈20 h) until complete dryness. Each dried ball‐mill product was transferred to Al‐crucibles, covered with crucible lids, and calcined in a box furnace at 950 °C (typically, three times) until the corresponding LLZO garnet phase was identified for each sample powder by XRD analysis. Of note, because the Al‐crucible may introduce aluminum into the powders, only powders from the center of the crucibles (not attached to the walls) were used for analysis by XRD, ICP‐MS, and calorimetry measurements. Sample powders were stored in a glovebox to prevent reaction between LLZO samples and ambient H_2_O and CO_2_.

### Structural characterization

Crystallographic parameters of the garnet powders were analyzed from PXRD measurements done with a Rigaku Ultima IV diffractometer using CuK_α_ (40 kV, 40 mA) as the radiation source. Prepared on low‐background silicon sample holders, their powder patterns were recorded at room temperature across 10°<2*θ*<50° with 0.02°, 2*θ*, and 1 s per step. Diffractogram peak pattern profiling and refinements (Match! 3.0, Crystal Impact) characterized and then quantified the structure phases and lattice parameters against the Inorganic Crystal Structure Database (ICSD).

### Composition analysis

Metals content in each sample was measured using ICP‐MS (Thermo X Series II). In brief, approximately 10 mg of each LLZO powder was dissolved in approximately 1 mL of aqua regia. Dissolution was rapid (<20 min.). These solutions were then twice diluted into 2 % HNO_3_ to give solutions containing approximately2.5–100 ng mL^−1^ of each element (Li, La, Zr, and Ga). Calibration standards were prepared in 2 % HNO_3_ by diluting certified single‐element plasma standards (1000 μg mL^−1^, VWR BDH, or Ricoh). The ICP‐MS analysis yielded a limit of detection (LOD) of 0.53(3), 0.084(1), 0.014(1), and 0.021(3) ng/mL for ^7^Li, ^139^La, ^90^Zr, and ^69^Ga, respectively. Measured concentrations of each isotope in the LLZO solutions were always approximately 10–1000 times greater than the reported LODs. Each dissolution and analysis were repeated in duplicate, yielding standard deviations in the calculated mol ratios of less than ∼2 %. The measured Zr content in each of the LLZO products was less than targeted (i. e., Zr apfu≈1.75 instead of 2.0). In agreement with the PXRD data, which provide no evidence of secondary phases within the LLZO products (with the exception of the product with the highest Ga content, as discussed above), the high precision and reproducibility of the measured Zr concentrations in individual samples and the similarity in measured Zr content between different LLZO products suggest that the Zr deficit does not result from secondary, insoluble Zr phases. Oxygen content was calculated from charge neutrality, assuming the LLZO structures contain only Li^+^, La^3+^, Zr^4+^, Ga^3+^, and O^2–^. The compositions were then normalized to La=3.00.

### Conductivity

Ionic conductivities were determined from electrochemical impedance spectroscopy (EIS) using a Solartron® 1260 Impedance Analyzer. Sample powders were pressed into 15 mm×1 mm (diameter×thickness) pellets with a uniaxial mechanical press (1400 psi) for 1 min. Both sides of the pellets were sputtered with a thin layer of Pt/Au coating as the electronic conductive interlayers between the pellets and instrument. The impedance of each sample pellet at room temperature (25 °C) was measured in the frequency range of 0.1 Hz to 10^6^ Hz in the EIS.

### Calorimetry

High‐temperature oxide melt solution calorimetry measurements were done with a twin Calvet‐type calorimeter (Setaram AlexSys) at 700 °C calibrated with the heat content of alpha‐alumina (Alfa Aesar, 99.999 %) using well‐established methods.^[64]^ Sample powders were hand‐pressed into pellets (nominally 5 mg) and dropped from room temperature into molten sodium molybdate (3Na_2_O ⋅ 4MoO_3_) contained in a platinum crucible housed in a quartz glassware assembly. The assemblage and molten salt solvent were flushed and bubbled continuously with oxygen (50 and 5.0 mL min^−1^). Flushing maintains the vapor pressure equilibrium above the solvent and bubbling aids in sample dissolution. The mean enthalpy of drop solution (Δ*H*_ds_) was computed from repeated measurements (total of 8–12 drops per sample) for which the mean value is reported as the 2‐sigma error.

## Conflict of interest

The authors declare no conflict of interest.
